# A Risk Exchange: Health and Mobility in the Context of Climate and Environmental Change in Bangladesh—A Qualitative Study

**DOI:** 10.3390/ijerph18052629

**Published:** 2021-03-05

**Authors:** Patricia Nayna Schwerdtle, Kate Baernighausen, Sayeda Karim, Tauheed Syed Raihan, Samiya Selim, Till Baernighausen, Ina Danquah

**Affiliations:** 1 Heidelberg Institute of Global Health, Heidelberg University, 69117 Heidelberg, Germany; kate.baernighausen@uni-heidelberg.de (K.B.); till.baernighausen@uni-heidelberg.de (T.B.); ina.danquah@uni-heidelberg.de (I.D.); 2 Nursing and Midwifery, Faculty of Medicine, Nursing and Health Science, Monash University, Clayton, VIC 3800, Australia; 3 School of Public Health, University of the Witwatersrand, Johannesburg 2000, South Africa; 4 Centre for Sustainable Development, University of Liberal Arts Bangladesh, Dhaka 1209, Bangladesh; sayeda.karim@ulab.edu.bd (S.K.); tauheed.raihan@gmail.com (T.S.R.); samiya.selim@ulab.edu.bd (S.S.)

**Keywords:** climate change, migration, health, healthcare

## Abstract

Background: Climate change influences patterns of human mobility and health outcomes. While much of the climate change and migration discourse is invested in quantitative predictions and debates about whether migration is adaptive or maladaptive, less attention has been paid to the voices of the people moving in the context of climate change with a focus on their health and wellbeing. This qualitative research aims to amplify the voices of migrants themselves to add nuance to dominant migration narratives and to shed light on the real-life challenges migrants face in meeting their health needs in the context of climate change. Methods: We conducted 58 semi-structured in-depth interviews with migrants purposefully selected for having moved from rural Bhola, southern Bangladesh to an urban slum in Dhaka, Bangladesh. Transcripts were analysed using thematic analysis under the philosophical underpinnings of phenomenology. Coding was conducted using NVivo Pro 12. Findings: We identified two overarching themes in the thematic analysis: Firstly, we identified the theme “A risk exchange: Exchanging climate change and health risks at origin and destination”. Rather than describing a “net positive” or “net negative” outcome in terms of migration in the context of climate change, migrants described an exchange of hazards, exposures, and vulnerabilities at origin with those at destination, which challenged their capacity to adapt. This theme included several sub-themes—income and employment factors, changing food environment, shelter and water sanitation and hygiene (WaSH) conditions, and social capital. The second overarching theme was “A changing health and healthcare environment”. This theme also included several sub-themes—changing physical and mental health status and a changing healthcare environment encompassing quality of care and barriers to accessing healthcare. Migrants described physical and mental health concerns and connected these experiences with their new environment. These two overarching themes were prevalent across the dataset, although each participant experienced and expressed them uniquely. Conclusion: Migrants who move in the context of climate change face a range of diverse health risks at the origin, en route, and at the destination. Migrating individuals, households, and communities undertake a risk exchange when they decide to move, which has diverse positive and negative consequences for their health and wellbeing. Along with changing health determinants is a changing healthcare environment where migrants face different choices, barriers, and quality of care. A more migrant-centric perspective as described in this paper could strengthen migration, climate, and health governance. Policymakers, urban planners, city corporations, and health practitioners should integrate the risk exchange into practice and policies.

## 1. Introduction

Under pessimistic and realistic scenarios of sustained high emissions and unequal development, climate change will represent an increasingly important factor contributing to internal migration [1,2]. In 2018, 17.2 million people were internally displaced due to natural disasters and extreme weather events [3], and in 2019, this rose to 24.9 million people [4]. Most internal displacement happens secondary to natural hazards and the majority occurs in Asia [3]. Despite the growing numbers of environmental migrants with the subset of migrants displaced for reasons related to climate change, definitions of “climate migrants” are still contested legally and academically and projections of migrant flows under different climate scenarios continue to be disputed.

Human mobility in the context of climate change refers to a range of responses from individuals, households, and communities, including migration, displacement, and planned relocation [5,6]. In Bangladesh, a great deal of this mobility is rural–urban, which is largely rapid and unplanned [7,8], setting up poor living conditions for migrants to survive and thrive in good health. 

Migration is considered an important determinant of health, and broadly speaking, migrants are considered a vulnerable group from a health perspective, because displacement can compromise other important determinants of health such as shelter, food, and water security and inclusion in labour markets, education, and healthcare services [1]. Our recent systematic literature review captured studies that examined diverse health issues associated with migration in the context of climate change including changing patterns of infectious disease, non-communicable disease, psychosocial conditions, and access to healthcare [9]. For these reasons and others, climate-related mobility is considered an issue of socio-political and humanitarian concern [6].

While there has been extensive research into the “dyad” research fields of climate change and migration and climate change and health, less is known about the climate change, migration, and health nexus [6,9,10]. The body of research on climate change, health, and migration variably characterizes climate migrants as a threat (securitization frame), victims (protection frame), and agents (migration as adaptation frame) [11]. Another way to summarise the research and discourse to date is that it a) explores perceptions of climate change and linking these to migration decision-making, b) projects migration flows under different climate scenarios, c) measures the extent to which migration acts as an adaptive or maladaptive response. While these insights are valuable for policy and practice, a deeper understanding is needed to broaden and diversify policy options regarding how to effectively protect and promote health and manage safe, orderly, and humane migration in the context of climate change [12]. 

**Figure 1:**
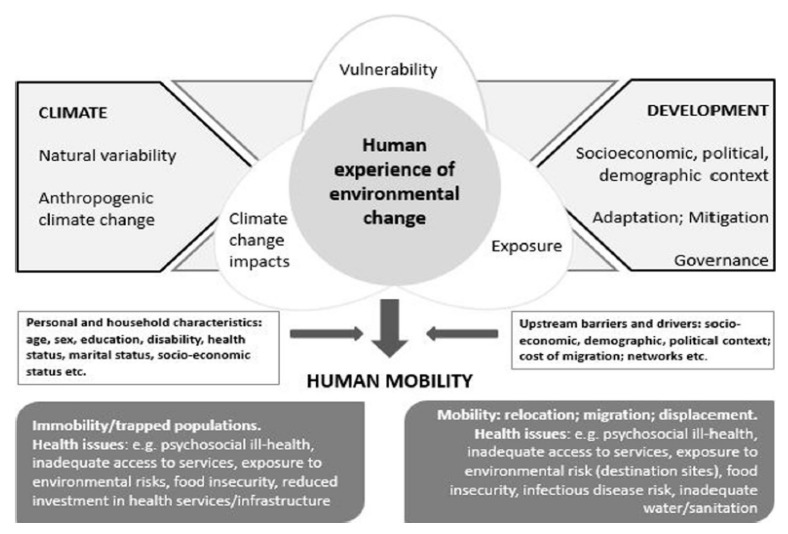
Conceptual framework of climate change and health risk. The relationship between climate change impacts, vulnerability, and exposure, and the links with human mobility and health outcomes [13,14]. Climate change impacts/Climate hazards: The potential occurrence of a natural or human-induced physical event or trend that may cause loss of life, injury, or other health impacts, as well as damage and loss to property, infrastructure, livelihoods, service provision, ecosystems, and environmental resources (Intergovernmental Panel on Climate Change (IPCC) Glossary). Exposure: The presence of people, livelihoods, species or ecosystems, environmental functions, services, and resources, infrastructure, or economic, social, or cultural assets in places and settings that could be adversely affected (IPCC Glossary). Vulnerability: The propensity or predisposition to be adversely affected. Vulnerability encompasses a variety of concepts and elements including sensitivity or susceptibility to harm and lack of capacity to cope and adapt (IPCC Glossary).

This paper focuses on the concept of climate risk, including specific risks for health due to climate change known as climate change and health risks. Climate risk is determined by the interaction between slow and sudden-onset hazards, exposure, and vulnerability and is depicted in Figure 1. Vulnerability, capacity, and adaptation assessments (VCAs) are an important tool to evaluate climate and health risk and explore strategies for prevention, enabling policymakers and practitioners to identify vulnerable groups, areas, and sectors and develop plans to mitigate risk and build resilience.

Bangladesh is one of the most climate-vulnerable countries in the world, due to the high population density with high levels of poverty in combination with a high reliance on natural resources and vast coastal areas [15,16]. Bangladesh experiences frequent and severe extreme weather events including flooding, cyclones and sea-level rise, and groundwater salinization. The Bangladeshi health system lacks the infrastructure, workforce capacity, and funding to fully address the additional load that both migration and climate change threaten to bring [15]. Bangladeshi people are highly exposed to climate hazards and often lack income diversification options, land ownership, or insurances. Further, exposure to slow-onset climate hazards can erode resilience over time, making disaster more likely when climate hazards strike [17,18,19].

This study aims to contribute to a deeper understanding of the role that socio-cultural factors play in the migration experience. We aim to contribute recommendations about how barriers to accessing healthcare may be reduced (downstream) and how the determinants of health might be optimized (upstream) to protect and promote migrant health and strengthen migration governance. Due to the complex and interacting drivers of migration, we do not seek to make causal inferences between climate change and health outcomes. Using qualitative methods, we sought to amplify the voices of migrants themselves to add nuance to dominant migration narratives and to shed light on the real-life challenges migrants face in meeting their health needs in the context of climate change.

## 2. Materials and Methods

In 2019, Bangladeshi researchers based at the University of Arts Bangladesh in partnership with researchers at Heidelberg University, Germany undertook a series of interviews with migrants living in Bhola slum in Dhaka, Bangladesh to explore their experiences of migration and health in the context of climate change. The research was interrupted by slum fires. Data collection was completed just before the COVID-19 pandemic reached Bangladesh and lockdowns were enforced, which led to significant migration back to origin.

### 2.1. Study Site

Bangladesh is a low-lying country in South Asia with an estimated population of 165 million [20], representing just over 2% of the global population and ranking as country number 8 after Nigeria in terms of population size [20]. As in many other developing countries, rural to urban migration contributes significantly to migration flows, with the rural population (as a percentage of the total population) rising from 8% (1974) to 23% (2001) to 36.6% (2018) to 39% today [21,22,23]. The country frequently experiences natural disasters including drought, heavy rain, and severe storm surges [24] contributing to decreased agricultural and fishery yields, which have in turn contributed to the migration to coastal and urban cities, which face their own climate change challenges [25]. 

This study was conducted in Dhaka city where approximately 6,970,105 people live within 1353 km^2^ [24,26,27]. Mirpur slum, also referred to as Bhola slum, is located in the mid-west of the capital of Bangladesh, Dhaka. Bhola slum is home to approximately 43,000 migrants (55.79% male, 44.21% female) [28]. Freshwater, sanitation, and healthcare facilities are scarce [29].

In Bangladesh, the health system and the underlying healthcare infrastructure are considerably underdeveloped and neglected [22]. The healthcare workforce is stretched with just 7.4 skilled health workers per 100 K people. Skilled health workers are defined as nurses, midwives, and physicians, and the recommended level according to the World Health Organisation (WHO) is 345 skilled health workers per 100 K people [30]. Reviews of the health system reveal corrupt and non-transparent practices and weak oversight mechanisms compromising the quality and safety of patient care [31]. There is no effective health information system, limited access to healthcare equipment and essential medicines, and large out-of-pocket expenses for patients [22]. The average health expenditure in South Asian countries and developing countries is 3.46 and 5.39% Gross Domestic Product (GDP), respectively. The most recent Bangladeshi budget increased allocation to the health sector to a mere 1.02% GDP [32]. The WHO sets 1.3% GDP as the benchmark to enable an adequate response to public health emergencies and to comply with International Health Regulations (IHR) guidelines [33]. Natural hazards and infectious disease outbreaks can pose shocks to the health system that can compromise core services.

### 2.2. Data Collection

In November 2019, four (two males, two females) Bangladeshi research assistants (RAs) were trained to collect qualitative data using standardized instruments, available in both Bangla and English. We piloted and revised instruments to refine translation, improve acceptability, and ensure cultural appropriateness. We employed purposeful sampling, using a screening tool to select participants whose migration decision was at least in part influenced by a sudden or slow-onset climate hazard. We obtained written informed consent from all study participants and audio-recorded one-on-one interviews in Bangla.

Socio-demographic data collected included sex, age, employment status, marital status, and religion. The interview guide used open questioning and used probing to follow up on issues that seemed relevant to participants or identified as important through the debriefing process. The questions sought to capture “what” migrants experienced concerning climate change, migration, and health and “how” they experienced it [34]. RAs also collected data in the form of reflexive and observational notes during and after the interviews. Interview duration ranged from 20–50 min. We conducted regular debriefing sessions with RAs and lead researchers throughout data collection to discuss and triangulate findings, amend interview guides, and refine lines of inquiry [35].

We simultaneously translated, transcribed, and checked interview transcripts for data accuracy by an external party. We anonymized identifying participant information and maintained confidentiality. We interviewed 60 adults (>18 years) men and women from migrant communities in Dhaka, Bangladesh, and 58 interview transcripts were included in this analysis. We excluded two interviews because one participant was determined not to be a migrant, and another interview was not audio recorded. The study received ethical approval from the Ethics Committee of the Medical School of Heidelberg University (S-928/2019) and the Ethics Committee of the University of Liberal Arts Bangladesh (OFR009).

### 2.3. Data Analysis 

Thematic analysis tenets guided the data analysis, which is a systematic approach to identifying themes through an inductive analysis of qualitative data [36]. We began our inductive approach during data collection with an analysis of notes from the daily debriefing sessions [36]. We identified several reoccurring themes during data collection and integrated them into the preliminary coding template. We expanded and restructured this template following line-by-line analysis of transcripts and via ongoing discussions between RAs and other authors regarding the presence and labelling of themes. We reviewed and refined reoccurring themes collaboratively using NVivo Pro 12 (QSR International, Melbourne, Australia) to manage and code the data.

## 3. Results

**Figure 2:**
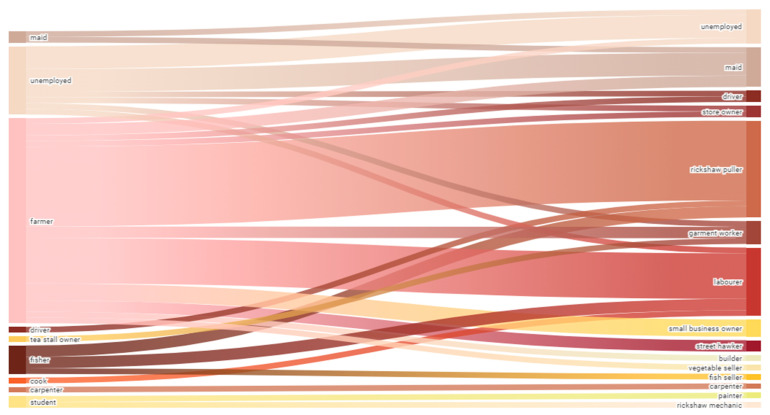
Change in employment pre-migration (origin) to post-migration (destination). Sankey Diagram: The left represents the occupations of participants before migration (at the origin in Bhola) and on the right, the occupations of participants post-migration (at destination in Dhaka). The width of the streams represents the number of participants involved in that transition. A few participants had more than one occupation at origin and destination, in which case, the transition stream was duplicated.

We interviewed 58 migrants whose decision to migrate to Dhaka was, at least in part, influenced by sudden or slow-onset climate hazards are included in this analysis. Table 1 presents the demographic characteristics of the participants. The migrants are predominantly male (M: 71%, F: 29%) and largely between 30 and 50, with a mean age of 43. While we tried to achieve an equal number of men and women, partners often prohibited women from participating in an interview, or it was felt that the male head of household would better represent their experience. We captured a range of migrants in terms of their length of stay in Dhaka, between 5 months and 33 years, with a mean of 17 years since the original migration. Of the participants who specified their educational background, most had no education (56%) or a primary level of education (36%). At origin, most participants were farmers, fishers, or unemployed, and many became indebted at least in part due to unfavourable farming conditions and weather and climate hazards. Most became rickshaw pullers, labourers, or maids at the destination. The women were mainly unemployed, farmers, or maids (domestic duties in their own home was counted as unemployed) at origin and became maids at the destination. The men were mainly farmers or fishers at origin and became rickshaw pullers or labourers at the destination. Figure 2 shows that migration led to employment diversification at destination. However, there were comparable levels of unemployment in both settings, and migrants described occupational health risks and exploitation at both origin and destination.

We identified two overarching themes from the thematic analysis. Firstly, we identified “a risk exchange of climate and health risk”. Migrants describe a changing environment in terms of threats and needs and requirement to adapt. Sub-themes under this theme include income and employment factors, changing food environment, shelter and water and hygiene sanitation (WaSH) conditions, social capital, and environmental determinants of health.

**Figure 3:**
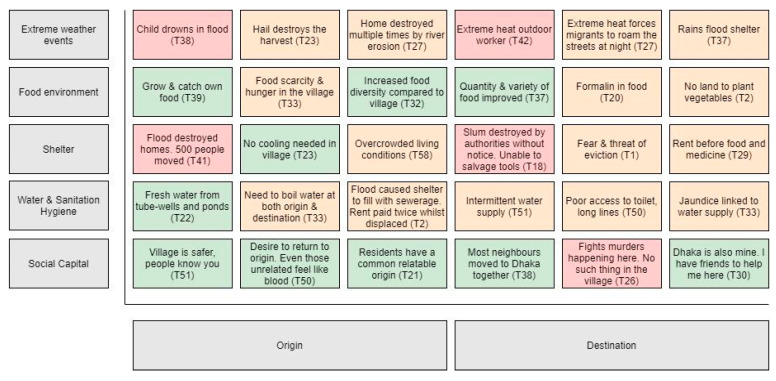
The climate change and health risk exchange at origin and destination. Heat map: This figure combines original data with an adapted version of a conceptual model by Warner et al. (2012) Figure 2: Household profiles affect whether migration is adaptive or erosive vis a vis rainfall, food, and livelihood insecurity.

The second overarching theme is the “changing health and healthcare environment”. Migrants described how their physical and psychosocial health changed over time with reference to their migration experience. This theme encompasses the following subthemes—physical and mental health status, healthcare as a business, navigating the health system, and the quality of healthcare. These two overarching themes were prevalent across the dataset, although each participant experienced and expressed them uniquely. We describe the themes and subthemes in detail below with quotes to substantiate the analysis (see Figure 3 for further quotes to support the themes).

### 3.1. A Risk Exchange: Exchanging Climate Change and Health Risks at Origin and Destination

The interviews reveal a complex risk exchange, whereby a range of challenges and opportunities at origin are exchanged for a different set of challenges and opportunities at the destination. The subthemes underneath this overarching theme related to pre-requisites for health and wellbeing, such as shelter and food, or social determinants of health, such as income and social capital.

#### 3.1.1. Risk Exchange Climate Change and Health Impacts at Origin and Destination

Migrants described direct climate change and health impacts at both origin and destination. At origin, they experienced floods, storms, and cyclones where people were injured, drowned, or experienced psychological trauma. In the crowded urban slum environment in Dhaka, extreme heat negatively affected the health and wellbeing of migrants.

- Extreme weather events and mortality: “*There is a lot, a lot of damage! Cows, goats, ducks, chickens, even people are swept away. There was a flood caused by a hurricane Nargis, even there was a flood 1 year ago there, people and people everywhere. At that time, someone suggested me to keep my hair tangled with a tree. People do not wash/flood away if they keep their hair tangled with trees*” (Transcript 25. 26 y.o female, referring to origin).
- Extreme weather and trauma: “*When the river erodes the homes they make this sound, like the sound of cattle dying as the water rushes against the house. The noise wakes up the residents and that is when they know the river is about to destroy their homes. They do not get much time to save their belongings*” (T22. 56 y.o female, referring to origin).
- Extreme heat: “*The children have to stay at home whole day, they do not get outside the home, we go out because we need to work, so they stay at home in hot whole day and they fall sick in extreme hotness. They got fever, diarrhoea or cough one or another disease they get*” (T58. 40 y.o male, referring to destination).

#### 3.1.2. Exchanging Risk—Income and Employment

Multiple drivers contributed to the migration decisions with different degrees of choice involved leading to different mobility responses including planned voluntary migration and forced displacement of individuals, households, and whole communities. Many migrants expressed a preference and inability to remain at origin (home).

Some migrants took loans to cover bad harvests and expressed a need to go to Dhaka to repay their debts after which time, most migrants intended to return home. However, many migrants seemed jarred by the very high cost of living in Dhaka and described livelihoods that were equally, if not more, fragile and insecure as those they experienced back home, where illness or injury could be devastating. Whilst many migrants described exhausting and hazardous working conditions at origin, many described high-risk working conditions and exploitation at the destination too (Dhaka).

- Exchanging income insecurity: “*It was really good there (origin). You cannot earn there. You can earn here (Dhaka) but you cannot eat here*” (T50. 65 y.o male).
- Difficult working conditions at the origin: “*(I wake up). around 4 to 5 am in the morning. After my morning prayer, I used to go to field. Several nights I could not even sleep because of the workload. Work, work, and work. (I go to sleep at) 3 am at 2 am. There was no fit time. I used to sleep 2–3 h. Like today I have to finish a particular … cultivation process but the labour cost is too high*” (T56. 60 y.o male, referring to origin).
- Difficult working conditions at destination: “*I wake up in the morning and go out to with rickshaw and come back within afternoon. I used to drive twice a day-morning and afternoon. But now I cannot, I pull it once in a day. My body does not support me to do that*” (T28. 50 y.o male, referring to destination).

#### 3.1.3. Exchanging Risk—Food Environment

Migrants described a changing food environment including improved food security where food was accessible, diverse, and nutritious, whilst others described a significant decline in food security with shortage and poor quality and safety. Concepts used by participants to describe a changing food environment included affordability, quantity, quality, accessibility, acceptability, taste, and safety.

- Quality and accessibility improved: “*The situation with the food was bad back in the village (origin). People would not ever be able to buy fish and here (destination) the market is close and we can buy the food and eat it*” (T10. 30 y.o female).
- Quality and safety worsened: “*Harmful..! There is not a single good food in Dhaka (destination). Frozen fish or ice-covered fish are available here. There is no such thing as fresh fish brought from the river at any time. Everything here is stale. In the village (origin), fish in rivers and vegetables can be found fresh or cultivated in the fields themselves*” (T26. 32 y.o male).
- Affordability worse: “*Back in our hometown (origin), we ate well. There we used to cultivate vegetables in our house compound but here in Dhaka (destination), we have to buy everything. Here we do not have the space to do these. We can’t even buy meat because of its high price*” (T19. 55 y.o female).

#### 3.1.4. Exchanging Risk—Shelter Environment

A key source of concern for migrants was insecure shelter expressed as concerns about paying rent and the continual fear of expulsion, abusive landlords, property destruction, and the high cost of living. A fire in the slums interrupted data collection and resulted in the secondary displacement of some migrant households.

- Property destruction by authorities: “*I am fine with them (authorities) destroying our homes, it being a government area, but give us a month to move. But they didn’t give us any warning, not even a second*” (T21. 60 y.o male, referring to destination).
- Secondary displacement: “*Authority demolishes that slum, so we relocate here…No, it’s not (life is not secure here). But we don’t have any other place to go. Everywhere is the same situation*” (T13. 24 y.o female, referring to destination).
- Insecure shelter: “*What to say! I have no peace from Dhaka city. The difference between Dhaka and one’s own village is like day and night. If the date crosses from 10th to 15th of the month, the landlord starts screaming and asks for house rent. If we are 2 days late, tell me to leave the house. If I have rented for 20 years, the landlords will say the same. And when I am in own home, there is no one to talk to like this*” (T1. 35 y.o female).

#### 3.1.5. Exchanging Risk—Water, Sanitation, and Hygiene (WaSH)

No participants described an improved water and sanitation environment post-migration. Key concerns included intermittent access to water, water scarcity, poor water quality, and the need to boil water in an environment where power was limited and expensive. Migrants also described competitive access to limited toilets with very poor hygiene.

- Poor water quality: “*The environment is polluted in Dhaka (destination). However, things were better in Bhola (origin). Here the water quality is really poor. It contains iron, sometimes smells bad, quite undrinkable. We have to refine it by boiling to drink it*” (T12. 28 y.o female).
- Limited sanitation facilities: “*This is a slum area, too many people live here, that’s why we have to stand in a line to go to the toilet*” (T26. 32 y.o male, referring to destination).

#### 3.1.6. Exchanging Risk—Social Capital

Social ties acted as a pull factor towards Dhaka and also kept people connected to their origin whilst working in Dhaka. Some participants described fragmentation of social ties and isolation in Dhaka, whilst those who migrated with other households or a bigger community described the maintenance or re-establishment of social ties that seemed to be linked with a more positive migration experience with possible implications for mental health and wellbeing.

- Social capital maintained/re-established at destination: “*I feel better here (destination) to be honest and here I have many friends and mates with whom I can share my problems or spend quality times*” (T55. 35 y.o male, referring to destination).
- Social capital decreases at destination: “*There is freedom in the village (origin). One could be known as someone in their own village but in Dhaka (destination) nobody knows you. We had no worries back in the village, but here we have a lot of worries*” (T20. 45 y.o male).

### 3.2. A Changing Health and Healthcare Environment

Participants discussed their mental health and wellbeing in terms of a lack of peace and overwhelming loss. They also described a changing healthcare environment with a range of services and models that seemed to require a high level of health literacy to navigate. Migrants experienced numerous barriers to accessing healthcare including financial and geographical barriers. Migrants contrasted the quality of care at origin with that at the destination and provided some valuable insights into what they considered quality healthcare to be.

#### 3.2.1. Changing Physical and Mental Health Status

Migrants gave accounts of a range of health problems they struggled to manage with the limitations of the health system at both origin and destination. Migrants described their health as deteriorating or remaining unchanged post-migration and often connected this assessment with environmental determinants of health such as air and water quality and working conditions.

- Health status deteriorating at destination: “*It (health) was good in the village (origin). In this place people are sicker, loud words, screaming, and slum’s disturbance, waste-garbage, uncountable mosquitoes*” (T1. 35 y.o female).
- Health status unchanged through migration: “*Diseases are always there, fever and body aches are always there. A constant. We have to take pills to work and live*” (T23. 45 y.o male, referring to destination).

Several participants subtly expressed mental health and wellbeing issues, speaking of a lack of peace often combined with a sense of loss that extended beyond material loss to social and cultural loss and damage. Loss was also expressed as a loss of identity, loss of family trade and inherited livelihood, loss of social ties, and a loss of connection to homelands.

- A lack of peace: “*When we lived in the village we lived so peacefully. Even if we do not eat one time we were in peace. Coming here we work, we eat but there is no peace. I keep thinking of the house rent, keep thinking how I will feed my children and do treatment*” (T58. 48 y.o male).
- Loss of land and connection to homeland: “*When we left our house, we broke down into tears... I thought if I pull rickshaw at least I would get money to feed my family. Our poor life is only for that. What I had lost it is unbearable. We are facing troubles while paying borrowed money*” (T33. 42 y.o male, referring to destination).
- Loss of identity: “*So the people in the village (origin) and in here (destination) are very different, the village area and town area very different. So we are people of village, when we come to the town, we become somebody else. That is why we go back to village to get well*” (T58. 40 y.o male).

Although some migrants periodically returned home to visit relatives, seek healthcare, or recover from illnesses, others described a willingness but inability to return for a visit. When discussing loss and mental health and wellbeing, some migrants talked about feeling trapped at their destination.

- Trapped at destination: “*Living in Dhaka is like living in a jail. But I still have to live here...It’s like taking poison. I have no other way that’s why I am here*” (T49. 45 y.o male, referring to destination).
- “*The village (origin) is a place for enjoyment. I used to enjoy myself there (origin). Even if I did not get food, I would be at peace. There is no peace here (destination). Here I am carrying out a sentence*” (T50. 65 y.o male).

Participants described a pervasive desire to return to origin, even though they admitted in the same interviews to not having a home, family, nor livelihood to return to. This desire seemed to be grounded in a connection to country/birth place. In other cases, the desire to return was conditional and depended on available housing and employment, and repayment of debt.

- Connection to country: “*After all, hometown (Bhola) is special. It is said that in a Bengali son, you cannot find such a homeland somewhere else, own homeland is the queen of all countries/places, it is own homeland. Hometown has no comparison*” (T27. 32 y.o male, referring to origin).

#### 3.2.2. Changing Healthcare Environment: Quality of Care and Barriers to Accessing Care

Migrants describe scarcity in terms of health services at origin, and difficulties in interacting with the health system at destination (Dhaka), experiencing mainly financial and geographical barriers to accessing healthcare. Some migrants described having to choose between eating and paying for medicine and that illness and injury can be bankrupting.

- Financial barriers: “*It cost 5000 taka to operate Caesar (caesarean section). Yet, Caesar was not done finely, there was problem with sewing. Then, we went to doctor for a solution, he asked for 2000 taka to do Caesar again. Don’t have to pay after that? We paid 1500 rupees after a lot of requests. Two stitches were opened, after paying he sewed finely again”* (T25. 26 y.o female, referring to destination).
- Illness can be devastating: “*Problems arrived one after another, my wife got sick. I spent around 3, 4 lacs for her surgeries, with my own earnings. After that I met the accident, again on my leg I spent around 2.5 lacs. However, I couldn’t buy medicine for myself, I wandered around asking for money from people. Not everyone could help, but some did and this is how I bought my medicines although I couldn’t cure my leg. It still hurts and I cannot move it”* (T18. 67 y.o male, referring to destination).

Many migrants used pharmacists as a first point of call for primary healthcare needs. Pharmacists also acted as a point of referral. Other health services, such as clinics and hospitals, were largely used for serious matters and emergencies. There were no accounts of using health services reactively, rather only preventatively. Similarly, migrants accessed health services for physical complaints—there were no accounts of migrants using health services for mental health issues. Linking to financial barriers to accessing care, some migrants described healthcare as just another business. When describing different levels of quality care at origin and destination, migrants referred to concepts of accessibility, affordability, and patient satisfaction.

- Healthcare as a business: “*They even ask for money for a normal vaccine. Fifty taka for a single one. No free medicine. They do not even talk without any money. What kind of healthcare is this?*” (T19. 55 y.o female, referring to destination).
- “*Yes, I take medicines they prescribe to me. They give out medicine depending on the money, the more money you have the more medicine they give you*” (T39. 40 y.o male, referring to destination).
- Quality of care: “*I would say Bhola’s (healthcare is better) because Bhola’s doctor didn’t give us several tests which cost us around 3–4 thousand. He simply gave us 2–3 medicines and that would do. Dhaka’s doctors do business more than treatment. I have pain in my back, my leg hurts and a part of my head hurts every now and then. But I do not visit the doctor in fear of tests and costly medicines*” (T18. 67 y.o male, referring to destination).
- “*The doctors here (Dhaka) do not understand. The doctors back home (Bhola) understand. They (Drs in Dhaka) will not listen to us at all. They only prescribe some random pill and tell us to pay them an absurd amount of money. They will tell us what we have before we explain it and make us visit again after we have taken the pills. The follow-up also costs a lot of money*” (T23. 45 y.o male, referring to destination).

Figure 3 is a type of heat map depicting the risk exchange migrants experienced in this study. The vertical axis depicts five categories of risk that migrants experienced at both origin and destination—extreme weather events, food environment, shelter, WaSH, and social capital. The horizontal axis shows whether the risk occurred at origin or destination. The coloured tiles within the heat map depict the codes that were identified in the interviews with reference to the transcript. The colours indicate the degree of risk to health from high (red), medium (orange), and low (green). The heat map presents the different threats and opportunities migrants experienced before and after migration concerning climate change and health. The codes were captured in the themes and explained further in the analysis and discussion.

## 4. Discussion

People who move in the context of climate change make complex decisions by weighing up changing vulnerabilities, exposures, and capacities where health is both a factor and an outcome of the migration decision and experience. The migrants in our study undertook a “risk exchange” concerning climate and health risks without a clear net gain or a net loss. Migrants moving in the context of climate change also enter a different healthcare environment where various barriers present themselves, to which they adapt and have views regarding the quality of healthcare.

Our findings support existing literature in three main ways. First, our findings variably depict migration as adaptive, a form of climate risk management that can reduce health risks [37], and as maladaptive, which can result in poorer health and other outcomes [38]. This is evident in the themes that indicate the environmental and social determinants of health changing both in ways that protect health (i.e., food environment improving) and ways that diminish health (i.e., living conditions are crowded and hot). The evidence to date paints environmental migrants as both vulnerable and in need of protection and as agents with inherent resilience [39]. Our findings reaffirm this complexity with both frames apparent in our interviews and analysis (see Figure 3). 

Second, our findings support claims that climate-related migration can lead to the disruption of social ties with potentially adverse consequences for mental health and wellbeing. At the same time, our findings also show that existing social ties can inform migration decisions and provide material and social resources that can benefit migrants’ health and wellbeing and may reduce mental health stressors associated with climate impacts and migration [40]. 

Third, our findings reveal that climate-related migration is associated with social and cultural loss and damage. Internal migrants are ultimately the responsibility of the state, yet from a broader perspective, developing countries have contributed least to the emissions their citizens are now paying severely for. Therefore, this social and cultural loss should be taken into account in the global loss and damage discourse and include non-economic damage in the form of health and wellbeing impacts [19].

Our findings differ in important ways from the current literature of migrant health in the context of climate change. First, it is challenging if not impossible to determine the primary driver for complex migration decisions that are multi-causal and dynamic [9,39]. This goes against the common assumption that climate migration is somehow exceptional and characteristically distinct from other forms of migration. This framing serves to problematize migration but also shapes research questions and methods that seek to quantify climate migration with Ricardian analysis that cross-references population datasets with climate data [41]. With this in mind, it is problematic to sort migrants into arbitrary, siloed categories that simplifies the migration experience. In this study, we conceptualized climate related migration as a socio-ecological phenomenon, rather than as a distinct group of migrants. Alternatively, it would be more appropriate to develop policy and services with needs as a primary consideration, taking into account how available resources can be most effectively invested. This aligns with calls to capture the complexities of climate mobilities and their interconnectedness with related policies such as health, indigenous rights, and development [39,42].

Second, a subset of the existing literature depicts climate mobilities as adaptation or maladaptation, implying a net gain or net loss scenario. Studies have sought to determine whether climate mobilities were successful or not, are cross-sectional, and overlook time scales (impacts over the short, medium, and long term) [9,39,42]. Others are unidimensional, examining one level at which the outcome is appraised (individual, household, community, or systems level) [9,39,42]. The adaptive vs. maladaptive frame does, to an extent, acknowledge a continuum of success and can add value to policy discussions; however, our interviews revealed a more complex risk exchange where a range of challenges and opportunities at origin were exchanged for a different set of challenges and opportunities at the destination with respect to health and health determinants. This raises questions about the continued utility of this frame and whether the research needs to advance to more strategic epidemiological questions that benefit participants more directly. For example, our study found health outcomes were determined by a collection of changes at the level of pre-requisites for health (such as shelter and food) and social determinants of health (such as income and social capital) providing some practical entry points for policy and practice considerations when it comes to protecting and promoting the health of migrant, host, and home communities. There is a growing consensus that further research on climate-related migration and health should avoid framing migration as a problem to be controlled [1,39]. Related to this is the notion that it is no longer appropriate to ask in a general sense and on a large scale, whether migration is a failure to adapt or adaptation, because the answer will vary in every case [42] and, perhaps, does not concretely or constructively inform public health responses.

Third, existing literature focuses on health-seeking behaviour and barriers and enablers to healthcare access, yet less attention has been paid to the quality of healthcare from a migrant perspective [1,6,39]. A patient-centred viewpoint would be useful in guiding policy and practice decisions, in terms of models of care and patient/family feedback mechanisms to ensure a more transparent and accountable healthcare system that is inclusive of migrants. Considering limited resources, high reliance on donor funds for public health activities in Bangladesh, and a need to serve sending, mobile, and host communities, at the very least, catastrophic health expenditures must be avoided. At best, migrants will be included in social protection and health insurance schemes and have access to affordable, appropriate, and safe universal basic healthcare.

Recognising changing health risks and the diverse health outcomes for migrants in the context of climate change, the main implications of this research for practice are to consider this complexity when planning health services and public health responses. Further research could appraise the association and degree to which some factors act protectively and thereby improve health outcomes (i.e., migrant integration into the workforce at destination) and how others act deleteriously (i.e., fracturing of social ties). Cost–benefit analytic studies could illuminate the best investments to improve migrant health taking into account the benefits migrants offer host communities (such as labour services) and the benefits they offer sending communities (such as the mobilization of remittances). 

This research reveals how migrants use formal health services for physical complaints, for emergencies and reactively (rather than preventatively), reinforcing the need to strengthen health systems, reduce barriers, reorientate towards prevention, and more broadly ensure health services are migrant-inclusive and climate-resilient [13]. Further research could incorporate holistic perspectives looking at the entire migration cycle, rather than focussing on mobile communities and, by doing so, identify synergies and triangulate ways in which the health can be protected and promoted at origin, return, and transit [6].

Our findings highlight that migrants suffer from mental health and psychosocial issues, yet do not seem to utilise health services for non-physical complaints. Considering this when developing healthcare services could increase health service utilisation, cultural appropriateness and, potentially, improve health outcomes. Community-based mental health services have consistently demonstrated improvements in migrant mental health outcomes [1] and may benefit the host community as well. Given migrants’ enduring willingness to return home and the sense of loss of identity that comes with forced displacement, future practice and research could explore how retaining and strengthening links to places of origin could serve to protect health and possibly preserve indigenous knowledge and connection to country. 

Our findings detail a complex health system that seems to require a relatively high level of health literacy to navigate. Further research could explore health literacy as a barrier to utilising health service access and utilisation and find practical ways (such as developing activities and tools) to either reduce the level of health literacy required to access services or increase the health literacy of migrants, or both. Further research could take a patient-rights approach to migrant health services and explore the components of quality of care from both a provider and patient perspective. This highlights how patient and practitioner rights and responsibilities may be clarified and aligned to both reduce barriers to accessing care and to improve the quality of care. Extending on this, there is a need to promote tenant and employee rights too to avoid internal migrant exploitation. 

Our research raises concern about protection gaps for internal climate migrants in terms of health but also in terms of land rights, tenant rights, and employment rights with evidence of exploitation surfacing in many interviews. This raises a point of concern for diverse stakeholders, given that all migrants have rights to the underlying social, political, economic, and cultural determinants of physical and mental health such as clean air and water and non-discriminatory treatment [1]. Government recognition of informal settlements is likely a critical and necessary step to improving the health of internal migrants. Research that is more interventional is required concerning urban (slum) health and could take into consideration the needs and concerns of the migrants described in this study. Our findings are based on the experiences of 58 rural–urban migrants in Bangladesh who moved, at least in part, due to climate and environmental changes into crowded urban settings. Some elements of our findings may be extended conceptually (rather that empirically) to individuals and communities experiencing similar vulnerabilities and exposures.

One novel finding of this research that we have not seen published elsewhere is the concept of trapped at destination. The literature documents “trapped populations”, who lack the resources, assets, or networks to enable migration, and also voluntarily “immobile populations”, who choose to remain for reasons of place attachment, sociocultural continuity, and values [5,39]. Both types of immobility refer to the origin or sending population; however, this research depicts the migration journey without a distinct beginning or end but rather as a continuum, incorporating secondary displacement and the will to return to origin despite significant adversity. Therefore, people can be trapped or immobile at their destination too, living in sites of similar or increased climate risk with consequences for health. Less attention has been paid to the health impacts of immobility, yet some researchers argue that immobile populations living in sites of climate vulnerability might experience adverse health impacts as a result of changes in water and food security, climate hazards, disease ecology, and the psychosocial impacts of disrupted livelihoods [6], which is supported by this research. 

## 5. Limitations

Our work has several limitations. First, the participants are unlikely to represent all migrants in Dhaka, and the experiences of migrants who move in the context of climate change may differ from those moving because of a different constellation of drivers and pull factors. Second, the selection process was imperfect. Given contested definitions of climate migrants, we simply sought to select migrants whose mobility was at least in part related to climate change from their own perspective. We do not seek to demonstrate causal attribution between climate related migration and health outcomes in this qualitative study. Third, the themes may over-represent male views, given our sample was not gender balanced and men preferred to represent the households’ views. Fourth, our cross-sectional approach depicts the memory of the experience at one point in time and could not take into account how this experience and perceptions may change over time.

## 6. Conclusions

Our results demonstrate how Bangladeshis engaging in internal climate mobilities in the context of climate change can undertake a “risk exchange” at origin and destination, whereby they exchange a range of hazards, exposures, and vulnerabilities at origin for a different set at their destination, requiring continual adaptation to reduce climate-related health risks. The experience of climate-related migration can also be understood as multi-staged and sometimes cyclical, rather than linear, whereby people can be trapped or immobile at their destination, as we already know they can be trapped or immobile at their origin. Social ties can be fractured by migration yet can also serve a protective role for migrant health and wellbeing.

Along with changing environmental and social health determinants is a changing healthcare environment where migrants face different choices and barriers, requiring a high level of health literacy to navigate an unfamiliar health system. We illuminate migrant views regarding the quality of care that could provide useful insights when developing health and migration policy, designing health services, and effective public health responses. This may involve building more climate-resilient and migrant-inclusive services, improving the cultural competency of health workers, and incorporating preventative and mental health activities into models of care. Further, our results highlight a need to look beyond environmental threats to social threats migrants face such as tenant and employee exploitation, which calls for a rights-based approach. Policymakers, city corporations, health practitioners, and urban planners should integrate the complexities of the risk exchange into their discourse and decision making and approach policy, practice, and further research with a migrant-centric and climate-aware perspective. Internal migrants are ultimately the responsibility of the state and should be incorporated into public protection and insurance schemes to, at a minimum, prevent catastrophic health expenditure and, ideally, achieve universal healthcare. Our findings add depth to the collective understanding of climate-related migration and health in that, from a migrant perspective, the experience is more complex than a “net gain” or “net loss” in terms of health and wellbeing. The experience and health outcomes depend on the circumstances migrants find themselves in with respect to health determinants. This may serve as a useful entry point for practice and policy decisions.

## Figures and Tables

**Table 1:** Participant demographic information (n: 58).

Age	<20	1
	20–29	5
	30–39	19
	40–49	17
	>50	16
Gender	Female	17
	Male	41
Time in Dhaka (years)	<5	13
	5–9	7
	10–14	5
	15–19	8
	>20	25
Formal education	Primary	13
	Secondary	1
	Religious	2
	None	20
	Not specified	22

## Data Availability

The data presented in this study are available on request from the corresponding author. The data are not publicly available due to privacy and ethical restrictions.
